# 

**Published:** 1907-10-05

**Authors:** 


### Resident Medical Officers' Department.

### NOTES, QUESTIONS, AND COMMENTS.

The editor of the Resident Medical Officers' Department of The Hospital invites contributions to this column both from resident medical officers and from other members of the profession. Short articles, notes, queries, or suggestions bearing upon this branch of medical practice will always receive careful consideration, and those that are suitable will be published in due course. Correspondence, short and to the point, is particularly invited, as by this means the value of the R.M.O. Department will be greatly increased.

Articles, whether in the form of letters to the editor or otherwise, should in no case exceed 600 words in length, and should, if possible, be shorter than this. Postcards with short notes, questions, or notifications of recent hospital appointments will be welcomed.

All communications for this column must be guaranteed by the name of the writer, which will not be published unless he expressly wishes it, and should bear the words "R.M.O. Department" on the envelope or the address side of the postcard.

Items of news from the resident officers' quarters of the principal hospitals, infirmaries, and asylums throughout the kingdom are invited, and, when of sufficient general interest, will be published.

The editor will also be pleased to receive and reply to confidential communications from resident medical officers and to consider any suggestions that may be made for the improvement of this department of The Hospital.

The Mayor of Battersea has forwarded a letter to the Secretary of the Metropolitan Hospital Sunday Fund in which he states that he has been requested by the "organised workmen of this borough to protest against the action of the Fund in withholding financial assistance from the Battersea Anti-Vivisection Hospital." "The hospital," the letter proceeds, "has done, and is doing, a great and good work in the borough."

THE HOSPITAL *October* 5, 1907.

*Name* ..........................................................

*Address* ..........................................................

..........................................................

This Coupon must accompany manuscript or contributions intended for The Hospital.

